# Comparison of Phenols Content and Antioxidant Activity of Fruits from Different Maturity Stages of *Ribes stenocarpum* Maxim

**DOI:** 10.3390/molecules23123148

**Published:** 2018-11-30

**Authors:** Yuwei Wang, Delin Qi, Shulin Wang, Xiaohai Cao, Ying Ye, Yourui Suo

**Affiliations:** 1 State Key Laboratory of Plateau Ecology and Agriculture, Qinghai University, Xining 810016, China; wangyuwei0507@163.com (Y.W.); delinqi@126.com (D.Q.); wangsl1970@163.com (S.W.); 2 College of Agriculture and Animal Husbandry, Qinghai University, Xining 810016, China; xnmd_zygc@163.com; 3 Northwest Institute of Plateau Biology, Chinese Academy of Sciences, Xining 810016, China

**Keywords:** *Ribes stenocarpum* Maxim, Phenols, Mature stages, Box-Behnken design method, Antioxidant activity

## Abstract

Differences in the content of nine phenols and the antioxidant capacity of *Ribes stenocarpum* Maxim (RSM) fruits at different stages of maturity were investigated, and the extraction process of polyphenols from RSM was also optimized using Box-Behnken design method. Results showed that the content of the nine phenols varied considerably at different ripening stages; catechin, chlorogenic acid, coumaric acid, and ferulic acid were abundant in immature fruits but decreased with fruit ripening, whereas the levels of rosemary acid and querctin acid were low in immature fruits and increased with time, reaching the highest value after the fruit was completely mature. The phenols extracted from RSM fruits possessed good antioxidant activities for effective and rapid scavenging of DPPH and ABTS free radicals, as well as intracellular ROS. Analysis of the phenols content at different maturity stages indicated that the unripe fruits had significantly higher polyphenols content than mature fruits. Consequently, unripe fruits possessed higher antioxidant activities. According to the overall results of the extraction process optimization, the selected optimal conditions for extracting polyphenols from RSM were as follows: extraction time, 95 min; solvent concentration, 60%; ratio of sample to solvent, 1:25.

## 1. Introduction

**Figure 1:**
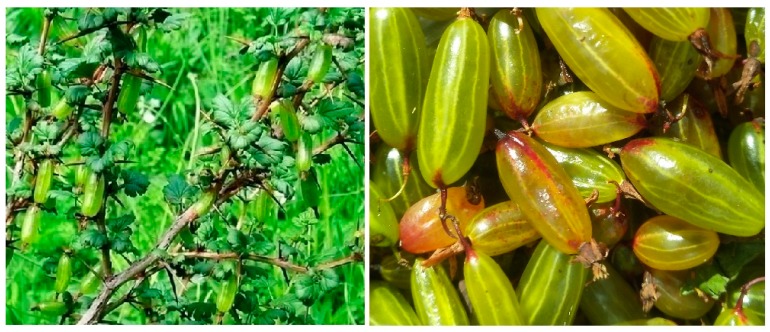
Fruits of *Ribes stenocarpum* Maxim.

*Ribes stenocarpum* Maxim (RSM, Figure 1) is a perennial deciduous shrub, found in Sichuan, Shanxi, Gansu, Qinghai, and other areas of China. There are approximately 160 species of *Ribes* plants worldwide, with 59 species and 30 varieties in China, and 11 species and one variety in Qinghai alone [1]. Reports show that the stems and branches of *Ribes* plants possess detoxification functions and can be used for the treatment of hepatitis [2]. In the Qinghai-Tibet plateau, the buds, flowers, and leaves of *Ribes* plants are brewed with boiling water as a tea drink, the fruits are boiled with water to cure colds, and the roots are used for treating rheumatism by the local people [3]. Fruits of *Ribes* plants, together with other immature wild fruits such as raspberries, blueberries, mountain grapes, and sea-buckthorn, also called third-generation fruits, are attracting increased attention because of their high nutritional value, strong health benefits, natural flavor, and absence of pesticide contamination [4,5,6].

Although *Ribes* plant resources are abundant in China, and RSM plants have been used as medicine against diseases such as common cold and rheumatism since antiquity in the Qinghai-Tibet plateau, studies on them are limited. The only related study analyzed the fatty acids contained in the seeds [7]. Currently, only blackcurrants of *Ribes* plants are in the limelight worldwide among the whole *Ribes* plant family. Slimestad [8] identified 15 anthocyanin glycosides from blackcurrant. Using the supercritical fluid extraction method, Sandell [9] extracted and identified more than a dozen flavonoids from the juice and residue of blackcurrant fruit, whereas Renata [10] obtained 18 phenolic acids from the fruit and leaves. Blech [11] also extracted and isolated trace amounts of β-cardoline alkaloids from the fruit of blackcurrant. Currently, extensive research in this field is underway.

Variation in plant species and the growth environment are important factors affecting the accumulation of chemical components [12,13,14]. The level of a component may vary by more than 10 times among species, showing large differences in biological activity [15,16,17]. Therefore, study of the phytochemical components of plants growing in specific environments such as the Qinghai-Tibet Plateau with high altitude and hypoxic climate is important. Plant polyphenols, which are secondary metabolic defense substances in plants, have attracted considerable attention for their unique biological activities such as strong antioxidant effects [18,19,20] and obvious anti-tumor [21,22,23], anti-viral [24,25,26], antibacterial [27,28], and anti-cholesterol effects [29,30]. Several experimental studies have shown that a certain amount of plant polyphenols can effectively prevent and inhibit the occurrence of diseases such as atherosclerosis [31,32]. In particular, the excellent antioxidant activities of plant’s polyphenols have triggered considerable interest in recent years. As natural antioxidants, these polyphenols are more resistant to free radical damage than most common synthetic antioxidants.

RSM, a member of the *Ribes* plant family widely distributed in the Qinghai-Tibet plateau, is rich in polyphenols. However, to the best of our knowledge, no systematic studies on the chemical composition and physiological activities of polyphenols from RSM of the Qinghai-Tibet Plateau have been reported [3]. To effectively use the more widely-distributed RSM resources in the Qinghai-Tibet Plateau, we procured the fruits of RSM as raw material in Qinghai, optimized the extraction process of polyphenols, determined the content of nine phenols of RSM fruits at different stages of maturity, and systematically investigated the antioxidant activity both in vivo and in vitro.

In this study, an efficient method for rapid extraction and separation of phenols was proposed. According to the response surface method, Box–Behnken design [33,34] was adopted to optimize several key parameters affecting extraction efficiency. The optimized methods were then applied for the analysis of nine phenols extracted from RSM fruits at different stages of maturity grown in the Qinghai-Tibetan Plateau. Accordingly, the antioxidant activities of phenols, including scavenging ability against 2,2-diphenyl-1-picrylhydrazyl (DPPH) and 2,2′-azino-bis(3-ethylbenzothiazoline-6-sulphonic acid) (ABTS) free radicals, and intracellular reactive oxygen species (ROS) using HepG2 cells as the model, were systematically investigated. This will provide a theoretical and scientific basis for the development and utilization of polyphenols from RSM fruits at different stages of maturity. In addition, this is the first report to date on the extraction, separation, and functional analysis of phenols from RSM fruits at different stages of maturity grown in the Qinghai-Tibetan Plateau.

## 2. Results and Discussion

### 2.1. Optimization of the Extraction Condition

To obtain optimal extraction conditions, a Box–Behnken design was used to optimize the significant variables (extraction time, solvent concentration, and ratio of sample to solvent), as well as to further investigate interactions between these variables. The experimental design for extraction process is shown in Table 1. According to the results of the Design Expert software, the P-value of regression model (P = 0.0002, < 0.05) indicated that the model is significant, whereas the P-value of lack-of-fit (P = 0.2333, > 0.05) indicated that, compared to pure error, the lack-of-fit is not significant. In this case B, C, A^2^, B^2^, and C^2^ show small P values (P < 0.05), i.e., the ratio of sample to solvent was the most significant variable for the model, followed by solvent concentration and extraction time. The empirical second-order polynomial model for the designs of extraction is shown in the following equation:

Y_1_ = +76.20 + 2. 27 * A + 6.31 * B + 8.17 * C − 1.26 * A * B + 0.19 * A * C + 2.26 * B * C − 5.53 * A^2^ − 10.92 * B^2^ − 10.52 * C^2^ (1)

**Figure 2:**
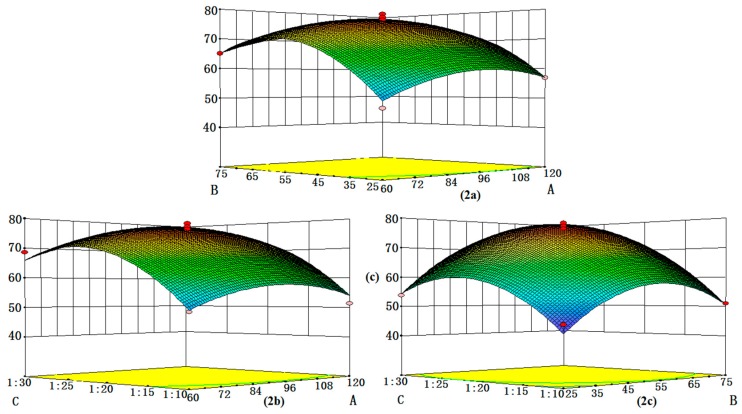
Response surfaces for the interactive effects of three variables on extraction efficiency of phenols from RSM fruits. (**a**). The combined effects of extraction time and solvent concentration. (**b**). The interactive effects of extraction time and ratio of sample to solvent. (**c**). The combined effects of solvent concentration and ratio of sample to solvent.

Based on the optimal conditions, three-dimensional response surfaces (cf. Figure 2a–c) were plotted to investigate the interactions among the variables and optimize each factor for the maximum content of phenols. According to the overall results of the optimization study, the selected optimal conditions were as follows: extraction time = 95 min; solvent concentration = 60% and the ratio of sample to solvent = 1:25.

### 2.2. HPLC Separation

**Figure 3:**
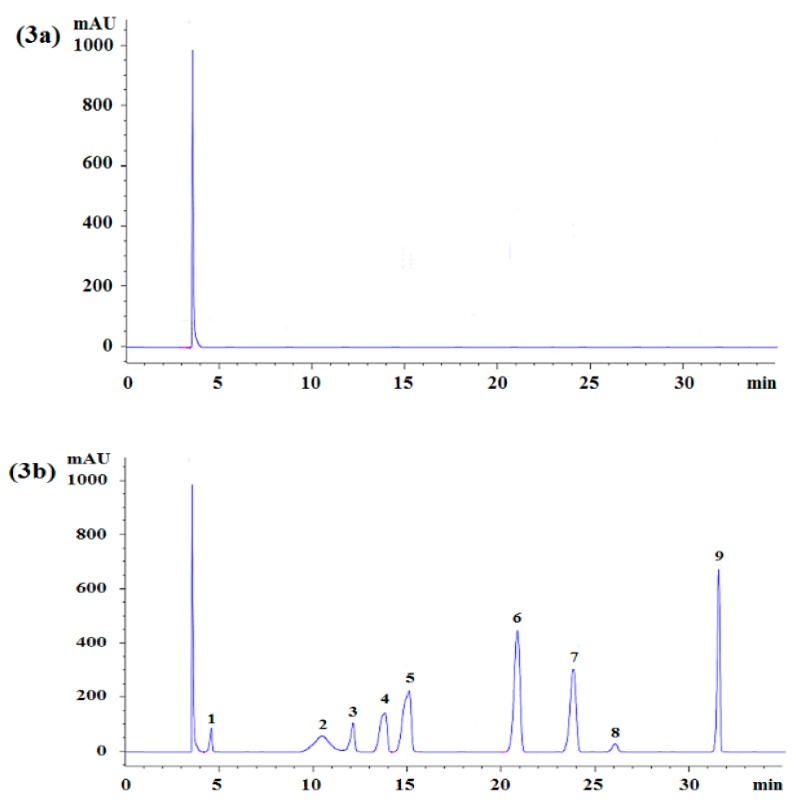

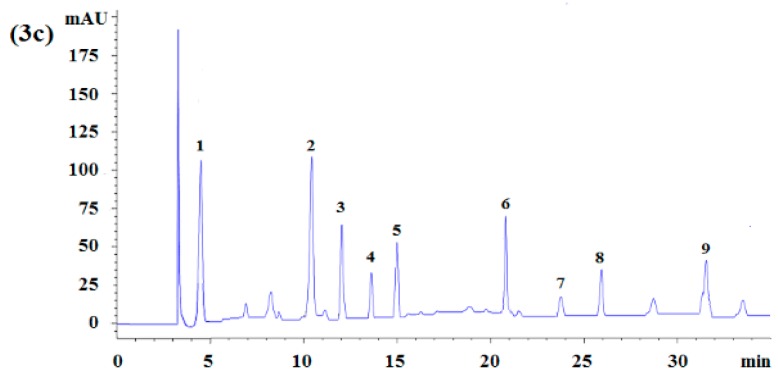
The representative chromatograms for blank (**a**), standards (**b**) and the typical chromatograms for the nine phenols in the sample of No. 5 (**c**). Peaklabels: 1. gallic acid, 2. catechin, 3. chlorogenic acid, 4. vanillic acid, 5. syringic acid, 6. cumaric acid, 7. ferulic acid, 8. rosemary acid, and 9. quercetin.

Results showed that gallic acid, catechin, chlorogenic acid, vanillic acid, syringic acid, coumaric acid, ferulic acid, rosemary acid, and quercetin were the main types of phenols in the fruits of RSM. These nine phenols were separated completely within 35 min with high resolution and symmetrical peaks by HPLC (Figure 3a–c). The HPLC chromatograms of blank sample, standard solutions, and the extracted samples are shown in Figure 3a–c.

### 2.3. Validation of the Method

The optimized method was validated for linearity, limits of detection (LODs), limits of quantification (LOQs), precision, and accuracy. Linearity data was generated by plotting the peak areas versus concentrations for the nine phenols standards, and are summarized in Table 2. The correlation coefficients were > 0.9969, indicating excellent linearity of the analytes. In addition, the LOD and LOQ ranges were from 0.10 to 0.32 ng/mL and 0.32 to 1.08 ng/mL, respectively. The precision of the instrument determined based on phenols was lower than 0.6 and 0.9 for the inter-day and intra-day validations, respectively (Table 2). All analyses were performed in triplicate. These results demonstrated that this method was a precise and practical method which is suitable for the determination of phenols extracted from the RSM.

### 2.4. Comparison of Phenols Content of RSM Fruits from Different Stages of Maturity

The established method and the optimal conditions were further applied for analyzing the nine phenols of RSM fruits from different stages of maturity. The composition of the nine phenols in dry materials were expressed as mean ± SD (n = 3) and are summarized in Table 3.

The results showed that the content of the nine phenols in the RSM fruits at different stages of maturity differed significantly. Catechin, chlorogenic acid, coumaric acid, and ferulic acid were abundant in immature fruits but decreased with maturity. Table 3 shows that the P-value of catechin content in sample 3–7 was less than 0.05, showing significant differences when compared to sample 1 (on August 10, 2016). Meanwhile, the highest levels of ferulic acid and coumaric acid were detected in the early fruits, reaching 24.17 mg/g (on August 10, 2016) and 23.07 mg/g (on August 10, 2016), respectively, then reducing rapidly in the 10-day interval. In particular, ferulic acid, compared with sample 1, showed a very significant difference. We speculated that the decrease in phenol content might be related to the instability of their structures, which had been converted into other substances during the biosynthesis process. The levels of rosemary acid and querctin acid were low in immature fruits and increased slowly with time, reaching the highest value after the fruit was completely mature. It is possible that their complex chemical structures contributed greatly to the slow process of biosynthesis in RSM plants. In addition, the contents of gallic acid, vanillic acid, and syringic acid reached the highest values in different semi-mature periods such as on September 1, September 20, and September 10, respectively; perhaps the change in enzyme activity during biosynthesis process in RSM plants was a relevant contributing factor, but the specific mechanism is unknown and needs to be further studied.

Currently, studies on phenols in RSM are limited. The reliable quantitative and qualitative determination of phenols composition and content determined in this study may lay a foundation for further investigation.

### 2.5. Analysis of Antioxidant Activities

#### 2.5.1. The DPPH and ABTS Free Radical Scavenging Activities

**Figure 4:**
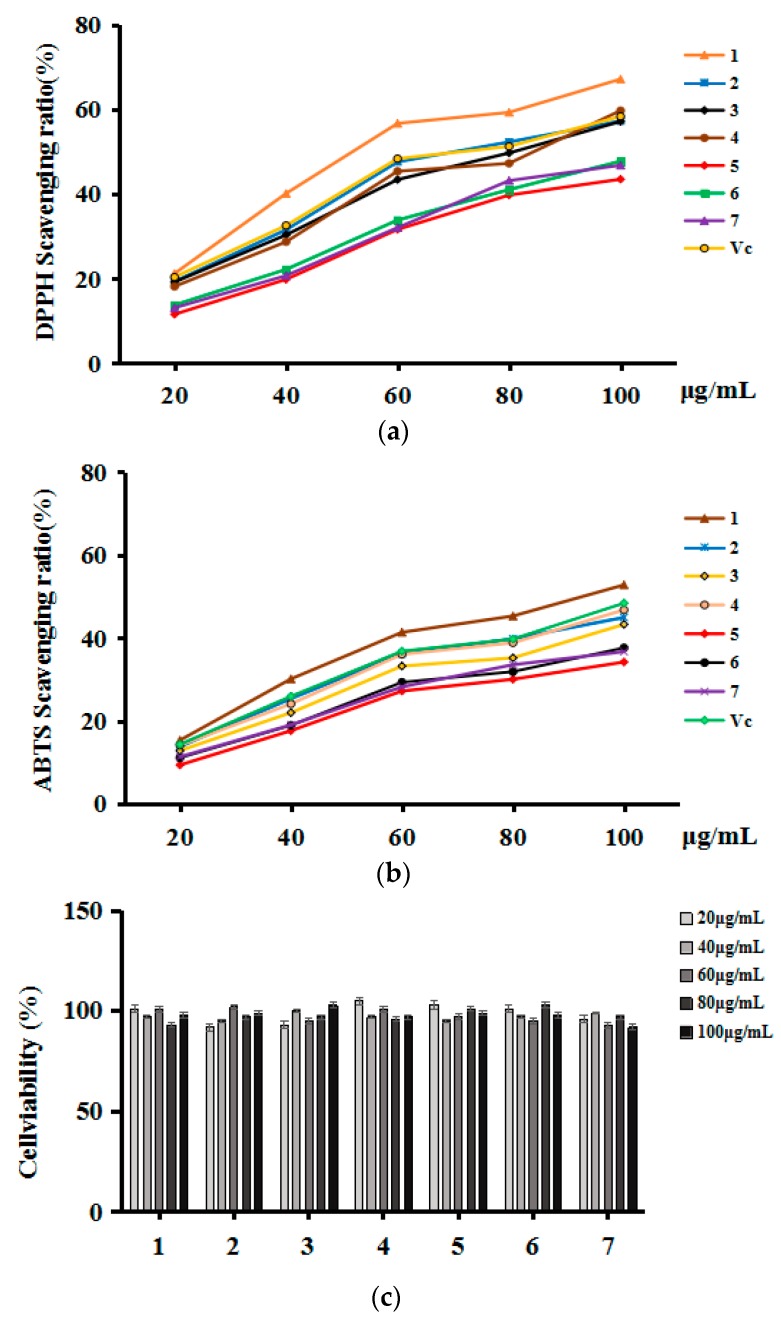

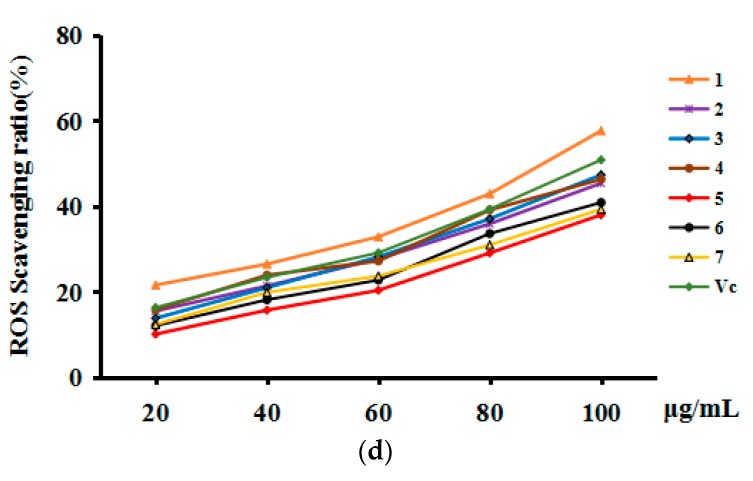
(**a**). Scavenging abilities of phenols from RSM fruits against the DPPH free radical. (**b**). Scavenging abilities of phenols from RSM fruits against the ABTS free radical. (**c**). Effect of phenols from RSM fruits on survival rate of HepG2 cells. (**d**). Scavenging abilities of phenols from RSM fruits against the intracellular ROS.

According to Figure 4a,b, the phenols extracted from RSM fruits at different stages of maturity possessed high antioxidant activities and effective and rapid scavenging abilities of DPPH and ABTS free radicals. In general, 100 μg/mL No. 1 sample showed scavenging ratios of up to 67.21% and 52.79% against DPPH and ABTS free radicals, respectively. Compared to Vc, the inhibition ability of DPPH and ABTS free radicals increased by 8.82% and 3.9% separately. The scavenging capacity of phenols from RSM fruits for DPPH successively ranged from high to low in the following order: No. 1, No. 4, No. 2, No. 3, No. 6, No. 7, and No. 5. The scavenging capacity of phenols for ABTS successively ranged from high to low in the following order: No. 1, No. 2, No. 4, No. 3, No. 6, No. 7, and No. 5. Compared to the other six samples, more coumaric acid and ferulic acid was contained in sample 1, and the inhibition activity of sample 1 on DPPH and ABTS free radicals was better. Maybe this indicates that coumaric acid and ferulic acid contributes significantly to the antioxidant activities of phenols extracted from RSM fruits. In addition, the inhibition ratio against DPPH free radical was significantly higher than that against the ABTS free radical, indicating that phenols scavenge liposoluble free radicals (DPPH) more than hydro-soluble free radicals (ABTS).

#### 2.5.2. Scavenging Abilities against Intracellular ROS

The toxic effects of RSM fruits at different stages of maturity on HepG2 cells were evaluated at concentrations ranging from 20 to 100 μg/mL to determine the safe dose for subsequent experiments. As shown in Figure 4c, none of the phenols extracted from RSM exhibited any significant effect on the viability of the HepG2 cells at the concentrations tested (P < 0.05). Thus, this concentration range can be considered safe for cellular experiments.

As shown in Figure 4d, the intracellular ROS scavenging abilities of the extracted phenols at concentrations ranging from 20–100 μg/mL showed a dose-dependent response. The phenols from sample No. 1 showed the highest ROS scavenging ability with the ratio of 57.63%, followed by No. 3, No. 4, No. 2, No. 6, No. 7, and No. 5, in which the ratios were 47.32%, 46.30%, 45.37%, 40.87%, 39.30%, and 37.98%, respectively. On the basis of these results, the scavenging abilities of RSM fruits at different stages of maturity can be ranked as No. 1, No. 3, No. 4, No. 2, No. 6, No. 7, and No. 5. In particular, the ROS removal capability of sample 1 was stronger in vivo than that of Vc, and the removal rate increased by 6.8% when the concentration was 100 μg/mL, which may also be related to the high content of coumaric acid and ferulic acid in sample 1. Of course, the specific reasons for this need to be further verified.

Overall, we concluded that antioxidant capacity was highest in the extracted phenols in the order No. 1 > No. 3 > No. 4 > No. 2 > No. 6 > No. 7 > No. 5. The antioxidant activity of RSM fruits decreased gradually with maturity, which may be due to the decrease in phenol content with the increase in fruit ripening. More importantly, all studies of antioxidant activity have shown that phenols in RSM fruits have good antioxidant activity and have the potential to develop into new antioxidants.

## 3. Experimental Section

### 3.1. Materials

The fruits of *Ribes stenocarpum* Maxim were collected from Huzhu in Qinghai province, China at different stages of maturity in 2017 (Table 4). The elevation of the picking land was 3029 m, longitude was 101° 50.926, and latitude was 36° 57.670.

### 3.2. Reagents

The nine phenols standards, including gallic acid, catechin, chlorogenic acid, vanillic acid, syringic acid, coumaric acid, ferulic acid, rosemary acid, and quercetin were of chromatographic grade and were purchased from Sigma Reagent Co. (USA). HPLC-grade acetonitrile and methanol were purchased from CLINC High Purity Solvents Co., Ltd. (Shanghai, China). Ultra-pure water was supplied by Watsons (Guangzhou, China). All other reagents used were of analytical grade unless otherwise stated.

### 3.3. Preparation of Standard Solutions

The stock solutions of the nine phenols were prepared in 90% acetonitrile, which were then used to prepare the mixed standard solution for HPLC separation (concentration of 1 × 10^−3^ mol/L), each of which contained dilutions of the stock solutions of the nine phenols with acetonitrile. All solutions were stored at 4 °C in the dark until analyzed.

### 3.4. Extraction

The RSM fruits were cleaned, air-dried, and milled. Because seeds were hard to crush, after passing through a 60 mesh sieve, seeds and pulp were separated, then all crushed pulp was collected for the next step of the test. During the extraction of phenols, 50 g of each sample was weighed out a brown bottle and then dissolved in 750 mL of 60% ethanol. The sample was ultra-sonicated at 45 °C for 95 min and 1.0 mL extracts were filtered through a 0.22 μm nylon syringe filter. The remainder was stored at 4 °C. All samples were measured under the optimized conditions of the previous process in triplicate.

### 3.5. HPLC Analysis

Studies show that gallic acid, catechin, chlorogenic acid, vanillic acid, syringic acid, coumaric acid, ferulic acid, rosemary acid, and quercetin are the main types of phenols in most plants [35,36,37], Therefore, the Hypersil™ GOLD (250 mm × 4.6 mm, 5 μm) column was used for the complete HPLC separation of the nine phenols, using acetonitrile-water with added formic acid as the mobile phase. The gradient involved an increase of solvent B (5% acetonitrile and 0.3% methanoic acid aqueous solution) in solvent A (0.3% methanoic acid aqueous solution) according to the gradient program: 0−8 min, 98%−95% B; 8−18 min, 95%−89.5% B; 18−21 min, 89.5%−89.5% B; 21−30 min, 89.5%−75% B; 30−35 min, 75−0% B. The injection volume was 10 μL and the flow rate 1.0 mL/min. In addition, prior to each analysis, the column was pre-equilibrated with the mobile phase for 5 min.

### 3.6. Experimental Design and Data Analysis

A Box-Behnken design was used for the optimization of extraction experiments, as a three-factor-three-level containing 17 experimental runs and total content of the nine phenols was considered as response values. Extraction time (X_1_), solvent concentration (X_2_) and ratio of sample to solvent (X_3_) were selected as the variables. In this experiment, the phenols extraction process was optimized taking the No. 5 sample as an example. The extraction of phenols from the other samples was undertaken using the selected optimal conditions. The experimental data was analyzed using Design Expert software (Version 7.1.6, Stat-Ease Inc., Minneapolis, MN, USA).

### 3.7. Biochemical Assays

The antioxidant activities of the phenols from RSM fruits were further assessed, which included scavenging activities against DPPH and ABTS free radicals, as well as intracellular ROS using HepG2 cells as the model. During preparation of the phenols extracts, the sample volumes were minimized to 50 mL in a vacuum at 45 °C. The phenol extracts were packed into a XAD-7 chromatographic column (4.0 cm × 30 cm) and adsorbed for 1 h. The column was eluted with acidified 1% ethanol at a flow rate of 2 mL/min and non-phenols substances were removed. The phenols were eventually eluted using acidified ethanol and concentrated in a vacuum at 45 °C, followed by freeze-drying to obtain phenol powders. The Sephadex LH20 dextran gel column (1.8cm × 80 cm) was equilibrated with phosphate buffered saline (PBS) pH 7.0. Then, 50 mg of the extracted phenols was eluted with PBS and the eluent was concentrated in vacuum, followed by freeze-drying to obtain the final extracted phenols samples. The samples were stored at 4 °C in the dark until analyzed.

#### 3.7.1. DPPH and ABTS Free Radical Scavenging Activity Assays

The DPPH-free radical scavenging activity of the extracts was based on a previously described procedure [38]. Vitamin C (Vc) solution of 0.5 mg/mL was used as the reference. The DPPH-free radical scavenging activity was expressed as scavenging ratio percentage calculated using the following equation:

Scavenging ratio of DPPH (%) = [1 − (A_sample_^1^ − A_blank_^1^)/A_control_^1^] × 100% (2)

where A_sample_^1^ is the absorbance of samples and DPPH solution mixture, A_blank_^1^ is the absorbance of the testing sample and absolute ethanol mixture, and A_control_^1^ is the absorbance of DPPH solution and solvent (i.e., distilled water or the corresponding buffer solutions) mixture.

ABTS free radical scavenging activity: This assay was performed as described previously [39] and absorbance values were measured using a spectrophotometer at 734 nm. Vc solution of 0.5 mg/mL was used as the reference. The antioxidant ability was calculated using the following equation:

ABTS scavengingratio (%) = [1 − (A_sample_^2^ − A_blank_^2^)/A_control_^2^] × 100% (3)

where A_sample_^2^ is the absorbance of testing sample, A_blank_^2^ is the sample background absorbance, and A_control_^2^ is the absorbance when sample is not present (i.e., buffer alone).

#### 3.7.2. Assay of Intracellular Activities of Phenols

Cell preparation: HepG2 cells were cultured in Dulbecco’s modified Eagle’s medium (DMEM)-containing 10% fetal calf serum (FCS) at 37 °C in a humidified incubator containing 5% CO_2_. The medium was replaced every other day until the cells were 80−90% confluent.

Cell toxicity assay: HepG2 cells in 150 μL growth medium were seeded in 96-well plates at 8 × 10^4^ cells per well in DMEM containing 10% FCS and incubated for 24 h. The cells were then treated with various concentrations of phenols for another 24 h. Thereafter, 10 μL MTT solutions (5 mg/mL) were added to each well and the cells were further incubated at 37 °C = b. After 4 h of incubation, the medium was removed and 150 μL dimethyl sulfoxide (DMSO) was added to dissolve the resultant formazan product. Absorbance was detected at 570 nm using a multifunctional enzyme-linked immunosorbent assay (ELISA) detection platform [40].

Intracellular ROS detection: HepG2 Cells were seeded in 96-well plates at a cell density of 8 × 10^4^ cells/mL for 24 h and treated with various concentrations of phenols for another 24 h. Then, the medium was replaced with 200 μL HBSS (Hank’s buffered salt solution) containing 25 μmol/L of 2′-7′-dichlorodihydrofluorescein diacetate (DCFH-DA) and further incubated for 1 h under the same condition. After that, the fluid was removed and the cells were washed thrice with HBSS solution. Then, the cells were treated with 100 μL AAPH (2,2′-azobis(2-amidinopropane) dihydrochloride) (0.6 mol/L) for 30 min in the CO_2_ incubator. The fluorescence intensities were measured using a multifunctional ELISA detection platform with the excitation and emission wavelengths of 485 and 530 nm, respectively [41].

## 4. Conclusions

In this study, an efficient method was developed for the rapid extraction and separation of nine phenols from RSM fruits at different stages of maturity. The optimum extraction conditions were as follows: extraction time, 95 min; solvent concentration, 60%; ratio of sample to solvent, 1:25. Analysis of the nine phenols from RSM fruits at different stages of maturity revealed that the content of these phenols varied considerably at different ripening stages. Catechin, chlorogenic acid, coumaric acid, and ferulic acid were abundant in immature fruits, but their levels decreased with fruit ripening; in contrast, the levels of rosemary acid and querctin acid were low in immature fruits and increased with time, reaching the highest value after the fruit was completely mature. In addition, the contents of gallic acid, vanillic acid, and syringic acid reached the highest values in different semi-mature periods. The antioxidative activities of these compounds were also investigated. Overall, analysis of phenol content at different maturity stages indicated that the unripe fruits had significantly higher levels than mature fruits. Consequently, unripe fruits possessed higher antioxidant activities, as observed in terms of the DPPH and ABTS free radical and intracellular ROS activities scavenging. However, our study has some limitations. As coumaric acid and ferulic acid were abundant in unripe fruits, we speculated that these acids contributed mainly to the antioxidant activity of polyphenols in RSM fruits. However, the phenols extracted from RSM fruits were not sufficiently pure, and antioxidant activity might improve with further purification of these phenols. Thus, the relationship between phenol species from RSM fruits and their antioxidant activities warrants further study. Overall, our work suggests that phenols from RSM fruits may act as new raw materials for the development of natural antioxidants. Our observations may further promote the use of *Ribes stenocarpum* Maxim resources, that are widely distributed in the Qinghai-Tibet plateau, for example, for the development of RSM-based functional food or medicine.

## Figures and Tables

**Table 1:** Numerical values of response obtained under the designed conditions taking the sample of No. 5 for example, and the contents of each phenol determined by HPLC as response values. (n = 3) (Supplementary Materials).

Run	Extraction Time (min)	Solvent Concentration (%)	Ratio of Sample to Solvent	Total Content of Nine Phenols (mg/g)
1	120.00	75.00	1:20	69.32
2	90.00	50.00	1:20	76.39
3	90.00	25.00	1:10	45.32
4	90.00	50.00	1:20	77.12
5	60.00	25.00	1:20	47.68
6	90.00	75.00	1:30	68.73
7	60.00	75.00	1:20	65.12
8	90.00	25.00	1:30	53.88
9	60.00	50.00	1:10	49.37
10	60.00	50.00	1:30	68.59
11	120.00	25.00	1:20	56.92
12	90.00	50.00	1:20	78.31
13	90.00	75.00	1:10	51.12
14	90.00	50.00	1:20	71.87
15	120.00	50.00	1:10	51.33
16	90.00	50.00	1:20	77.32
17	120.00	50.00	1:30	71.32

**Table 2:** Linear regression equation, correlation coefficients, LODs, LOQs, reproducibility of retention time and peak area, intra- and inter-day precisions.

Phenolic Acids	Regression Equationa	r	LOD	LOQ	Instrument Precision (n = 6)		Method Precision (n = 3)	
			(μg/L)	(μg/L)	Intra-Day	Inter-Day	Intra-Day	Inter-Day
Gallic acid	y = 0.853x − 0.042	0.9973	0.27	0.98	0.7	1.2	1.3	2.8
Catechin	y = 4.017x − 0.315	0.9981	0.32	0.95	0.6	0.9	1.4	2.7
Chlorogenic acid	y = 1.823x − 0.032	0.9978	0.28	1.08	0.6	1.1	1.9	3.7
Vanillic acid	y = 4.107x − 0.057	0.9992	0.27	0.77	0.8	1.1	1.3	2.4
Syringic acid	y = 7.154x − 0.127	0.9983	0.29	0.83	0.8	1.0	1.6	3.2
Coumaric acid	y = 9.987x − 0.141	0.9976	0.16	0.61	0.9	1.4	1.7	3.5
Ferulic acid	y = 7.357x − 0.068	0.9985	0.21	0.72	0.8	1.3	1.6	3.2
Rosemary acid	y = 0.669x − 0.013	0.9969	0.30	1.01	0.6	1.1	1.3	2.5
Quercetin acid	y = 6.830x − 0.063	0.9982	0.10	0.32	0.7	1.1	1.3	2.7

**Table 3:** Content of nine phenols of RSM fruits from different maturity stages (mean ± SD, mg/g, n = 3).

Phenols	Sample 1	Sample 2	Sample 3	Sample 4	Sample 5	Sample 6	Sample 7
Gallic acid	9.76 ± 1.20	8.53 ± 1.56	14.77 ± 1.98**	11.52 ± 1.88	10.13 ± 1.71	10.32 ± 1.80	7.02 ± 1.05
Catechin	19.71 ± 2.35	17.62 ± 2.13	16.15 ± 2.01*	14.10 ± 1.97**	13.33 ± 1.92**	11.09 ± 1.73**	8.59 ± 1.30**
Chlorogenic acid	14.21 ± 1.97	13.77 ± 1.93	12.17 ± 1.82	13.76 ± 1.95	9.49 ± 1.59**	5.32 ± 1.07**	2.15 ± 0.59**
Vanillic acid	1.35 ± 0.23	1.97 ± 0.28	3.11 ± 0.47**	4.53 ± 0.60**	5.18 ± 0.81**	5.03 ± 0.79**	4.81 ± 0.71**
Syringic acid	2.15 ± 0.46	5.19 ± 0.98**	7.17 ± 1.21**	11.36 ± 1.72**	7.30 ± 1.38**	6.09 ± 1.18**	3.12 ± 0.46
Coumaric acid	23.07 ± 2.98	18.65 ± 1.92*	14.73 ± 1.61**	12.90 ± 1.42**	10.00 ± 1.18**	13.19 ± 1.47**	15.77 ± 1.72**
Ferulic acid	24.17 ± 2.73	16.98 ± 1.77**	10.52 ± 0.95**	5.65 ± 0.56**	3.53 ± 0.65**	3.10 ± 0.43**	1.72 ± 0.29**
Rosemary acid	-	1.03 ± 0.27	1.79 ± 0.38	3.39 ± 0.76**	5.48 ± 0.89**	9.16 ± 1.32**	9.55 ± 1.28**
Quercetin acid	-	2.15 ± 0.31	2.97 ± 0.60	4.13 ± 0.87	6.40 ± 0.94**	7.11 ± 1.54**	11.39 ± 1.83**

1. Data are expressed as mean value ± S.D. 2. - Not detected.3. * *P* < 0.05, ** *P* < 0.01, control group: Sample 1.

**Table 4:** Samples of RSM from different stages of maturity.

Codes	Sample 1	Sample 2	Sample 3	Sample 4	Sample 5	Sample 6	Sample 7
Picking time	August 10, 2017	August 20, 2017	September 1, 2017	September 10, 2017	September 20, 2017	October 1, 2017	October 10, 2017

## Supplementary Materials

The following are available online.
