# Supplementary material for: Comparison of Phenols Content and Antioxidant Activity of Fruits from Different Maturity Stages of Ribes stenocarpum Maxim
Source: Molecules. 2018 Nov 30;23(12):3148. doi: 10.3390/molecules23123148 (PMC6321012; doi:10.3390/molecules23123148)
Supplement: Supplementary file 1 [file molecules-23-03148-s001.pdf]

Table 1 Numerical values of response obtained under the designed conditions taking the sample of NO.5 for example, and the contents of each phenols determined by HPLC as response values. (n = 3).

Run	extraction time (min)	solvent concentration (%)	ratio of sample to solvent	total phenolic acid content (mg/g)
1	120.00	75.00	1:20	69.32
2	90.00	50.00	1:20	76.39
3	90.00	25.00	1:10	45.32
4	90.00	50.00	1:20	77.12
5	60.00	25.00	1:20	47.68
6	90.00	75.00	1:30	68.73
7	60.00	75.00	1:20	65.12
8	90.00	25.00	1:30	53.88
9	60.00	50.00	1:10	49.37
10	60.00	50.00	1:30	68.59
11	120.00	25.00	1:20	56.92
12	90.00	50.00	1:20	78.31
13	90.00	75.00	1:10	51.12
14	90.00	50.00	1:20	71.87
15	120.00	50.00	1:10	51.33
16	90.00	50.00	1:20	77.32
17	120.00	50.00	1:30	71.32

Table 2 Linear regression equation, correlation coefficients, LODs, LOQs, reproducibility of retention time and peak area, intra- and inter-day precisions.

Analyte	Regression equationa	r	LOD	LOQ	Instrument precision (n=6)		Method precision (n=3)	
			(μ g/L)	(μ g/L)	Intra-day	Inter-day	Intra-day	Inter-day
Gallic acid	y=0.853x-0.042	0.9973	0.27	0.98	0.7	1.2	1.3	2.8

Catechin	y=4.017x-0.315	0.9981	0.32	0.95	0.6	0.9	1.4	2.7
Chlorogenic acid	y=1.823x-0.032	0.9978	0.28	1.08	0.6	1.1	1.9	3.7
Vanillic acid	y=4.107x-0.057	0.9992	0.27	0.77	0.8	1.1	1.3	2.4
Syringic acid	y=7.154x-0.127	0.9983	0.29	0.83	0.8	1.0	1.6	3.2
Coumaric acid	y=9.987x-0.141	0.9976	0.16	0.61	0.9	1.4	1.7	3.5
Ferulic acid	y=7.357x-0.068	0.9985	0.21	0.72	0.8	1.3	1.6	3.2
Rosemary acid	y=0.669x-0.013	0.9969	0.30	1.01	0.6	1.1	1.3	2.5
Quercetin acid	y=6.830x-0.063	0.9982	0.10	0.32	0.7	1.1	1.3	2.7

Table 3 Main content of phenols of RSM fruits from different maturity stages (mean±SD, mg /g, n=3).

**Samples**	**1**	**2**	**3**	**4**	**5**	**6**	**7**
Gallic acid	9.76±1.20	8.53±1.56	14.77±1.98	11.52±1.88	10.13±1.71	10.32±1.80	7.02±1.05
Catechin	19.71±2.35	17.62±2.13	16.15±2.01	14.10±1.97	13.33±1.92	11.09±1.73	8.59±1.30
Chlorogenic acid	14.21±1.97	13.77±1.93	12.17±1.82	13.76±1.95	9.49±1.59	5.32±1.07	2.15±0.59
Vanillic acid	1.35±0.23	1.97±0.28	3.11±0.47	4.53±0.60	5.18±0.81	5.03±0.79	4.81±0.71
Syringic acid	2.15±0.46	5.19±0.98	7.17±1.21	11.36±1.72	7.30±1.38	6.09±1.18	3.12±0.46
Coumaric acid	23.07±2.98	18.65±1.92	14.73±1.61	12.90±1.42	10.00±1.18	13.19±1.47	15.77±1.72
Ferulic acid	24.17±2.73	16.98±1.77	10.52±0.95	5.65±0.56	3.53±0.65	3.10±0.43	1.72±0.29
Rosemary acid	-	1.03±0.27	1.79±0.38	3.39±0.76	5.48±0.89	9.16±1.32	9.55±1.28
Quercetin acid	-	2.15±0.31	2.97±0.60	4.13±0.87	6.40±0.94	7.11±1.54	11.39±1.83

1. Data are expressed as mean value ± S.D.

2. -Not detected.

Table 4. Samples of RSM fruits from different stages of maturity

Codes	Sample 1	Sample 2	Sample 3	Sample 4	Sample 5	Sample 6	Sample 7
Picking time	August 10, 2017	August 20, 2017	September 1, 2017	September 10, 2017	September 20, 2017	October 1, 2017	October 10, 2017

Table 5. Determination of DPPH radical scavenging ability

μg · mL -1	Sample 1	Sample 2	Sample 3	Sample 4	Sample 5	Sample 6	Sample 7	Vc
20	21.21%	19.32%	19.21%	18.15%	11.57%	13.76%	13.11%	20.36%
40	40.15%	31.57%	30.35%	28.72%	19.83%	22.19%	20.72%	32.58%
60	56.73%	47.62%	43.44%	45.41%	31.65%	33.85%	32.07%	48.39%
80	59.34%	52.33%	49.73%	47.28%	39.77%	41.07%	43.19%	51.29%
100	67.21%	57.30%	57.19%	59.76%	43.52%	47.79%	46.83%	58.39%

Table 6. Determination of ABTS radical scavenging ability

μg · mL -1	Sample 1	Sample 2	Sample 3	Sample 4	Sample 5	Sample 6	Sample 7	Vc
20	15.32%	13.76%	12.81%	14.10%	9.35%	11.08%	11.33%	14.33%
40	30.11%	25.37%	21.97%	24.09%	17.62%	18.92%	19.02%	25.92%
60	41.35%	36.19%	33.21%	36.05%	27.19%	29.30%	28.19%	36.81%
80	45.28%	39.80%	35.18%	38.83%	30.03%	31.86%	33.53%	39.72%
100	52.79%	44.94%	43.27%	46.76%	34.18%	37.60%	36.69%	48.39%

Table 7. Effect of phenols from RSM fruits on survival rate of HepG2 cells

μg · mL $^{-1}$	Sample 1	Sample 2	Sample 3	Sample 4	Sample 5	Sample 6	Sample 7
20	101%	92%	93%	105%	103%	101%	96%
40	97%	95%	100%	97%	95%	97%	99%
60	101%	102%	95%	101%	97%	95%	93%
80	93%	97%	97%	96%	101%	103%	97%
100	98%	99%	103%	97%	99%	98%	92%

Table 8. Scavenging abilities of phenols from RSM fruits against the intracellular ROS.

μg · mL $^{-1}$	Sample 1	Sample 2	Sample 3	Sample 4	Sample 5	Sample 6	Sample 7	Vc
20	21.57%	15.51%	13.82%	15.80%	10.10%	12.06%	12.36%	16.29%
40	26.49%	21.39%	20.97%	23.91%	15.72%	18.15%	19.87%	23.38%
60	32.86%	27.68%	28.15%	27.19%	20.35%	22.75%	23.69%	29.12%
80	42.91%	35.89%	37.07%	39.11%	29.07%	33.61%	30.96%	39.29%
100	57.63%	45.37%	47.32%	46.30%	37.98%	40.87%	39.30%	50.83%
